# Engaging the hearts and minds of clinicians in outcome measurement – the UK rehabilitation outcomes collaborative approach

**DOI:** 10.3109/09638288.2012.670033

**Published:** 2012-04-16

**Authors:** Lynne Turner-Stokes, Heather Williams, Keith Sephton, Hilary Rose, Sarah Harris, Aung Thu

**Affiliations:** ^1^King’s College London School of Medicine, Department of Palliative Care, Policy and Rehabilitation, London, UK; ^2^Regional Rehabilitation Unit, Northwick Park Hospital, Middlesex, UK

**Keywords:** Rehabilitation, outcome assessment, clinical involvement

## Abstract

**Purpose:**

This article explores the rationale for choosing the instruments included within the UK Rehabilitation Outcomes Collaborative (UKROC) data set. Using one specialist neuro-rehabilitation unit as an exemplar service, it describes an approach to engaging the hearts and minds of clinicians in recording the data.

**Key messages and implications:**

Measures included within a national data set for rehabilitation should be psychometrically robust and feasible to use in routine clinical practice; they should also support clinical decision-making so that clinicians actually want to use them. Learning from other international casemix models and benchmarking data sets, the UKROC team has developed a cluster of measures to inform the development of effective and cost-efficient rehabilitation services. These include measures of (1) “needs” for rehabilitation (complexity), (2) inputs provided to meet those needs (nursing and therapy intervention), and (3) outcome, including the attainment of personal goals as well as gains in functional independence.

**Conclusions:**

By integrating the use of the data set measures in everyday clinical practice, we have achieved a very high rate of compliance with data collection. However, staff training and ongoing commitment from senior staff and managers are critical to the maintenance of effort required to provide assurance of data quality in the longer term.

## Introduction and background

In this era of evidence based practice, increasing attention is focussed on the outcomes of intervention. Outcome measurement in rehabilitation is required for research and clinical learning to support communication between clinicians and their patients and to convince purchasers that rehabilitation can be not only effective but also value for money. The need to measure outcome is therefore undisputed. The more challenging questions are as follows: (1) What to measure? (2) How and when to measure it? and (3) What to do with the data? Above all, though, the challenge is how to engage clinicians to record outcomes consistently.

**Implications for Rehabilitation**

- With increasing attention focussed on the outcomes of rehabilitation interventions, the challenge remains as to how to engage clinicians to record outcomes consistently.
- This article describes the development and adoption of the UK rehabilitation outcomes collaborative (UKROC) data set in one exemplar setting.
- User friendly software, embedding the use of tools into everyday clinical decision making and ongoing commitment and leadership from senior staff and managers appear to play a key role in getting buy-in from clinicians.

A wide range of standardised outcome measurement tools has been available for rehabilitation for over 30 years, so why do some clinicians use them and others do not? A number of authors have explored the factors that influence the implementation of standardised outcome measurement in rehabilitation and related settings. Taken together, this literature suggests that the principal barriers relate to practical difficulties – including time pressures, the burden of excess paperwork, and financial constraints (e.g. lack of support for data entry [1–4]). Other concerns relate to utility – the measures are not perceived to be helpful, or not to capture the important effects of treatment [1,2,4]. A third problem is lack of “know-how” – clinicians lack training in how to implement the tools or how to interpret the data [1,2].

On the positive side, standardised outcome measurement tools are more likely to be taken up where clinicians perceive them to be clinically useful [2]; for example, guiding clinical decision-making, monitoring progress or communicating with other teams or with the patient and family.

- In order to enhance uptake, other authors have emphasised the need for a compendium or basket of tools to be available [5,6] so that clinicians may choose (from a limited range) the instruments which best capture the intended outcomes in any given context.
- Both clinician-rated and patient-related tools should be available to capture impact on the individuals daily life (e.g. activities, work, social engagement) as well as change at impairment level [7].

Other key influences, however, include the payer and the work setting [2]. Outcome measurement is more likely to be implemented if the institution is committed to ensuring that it happens (by providing leadership and resources), or if a third party payer demands outcome reporting as part of the commissioning contract.

In the United Kingdom, a national clinical database for rehabilitation has recently been set up to record activity and outcomes for specialist rehabilitation services [8]. The data set takes a novel approach to the recording of outcome for the purposes of benchmarking and evaluation. This article explores the rationale for choosing the instruments included within the data set. Using our rehabilitation unit as an exemplar service, we describe an approach to engaging the hearts and minds of clinicians in recording the data – so that outcome measurement is seen, not as just another chore, but as an integral part of their daily practice.

### Key criteria for an acceptable outcome measure

The important psychometric criteria for a good measuring tool are well defined. It should be valid, reliable, reproducible, and have good scaling properties – ideally at interval level. However, as high-lighted by Alvan Feinstein in the late 1980s, if the tool is going to be widely taken up in clinical practice, it must also be “clinically sensible” – that is, it must be relevant, interpretable, responsive to clinically important change and feasible to apply in the clinical setting. Feinstein coined the term “Clinimetrics” and identified a certain tension between “standardisation” and “sensibility” [9].

- The psychometric approach to development of measuring tools emphasises the scaling properties of the tool and expects unidimensionality, but may not capture all the relevant information that clinicians needs to know.
- The clinimetric approach accepts that some clinically useful outcome measures may not have such good scaling properties, but may nevertheless capture the critical information to distinguish between a good and a poor outcome. Multi-dimensionality is therefore not only tolerated but also expected.

Nevertheless, if the tool is to be used to provide quantitative data, it is important to understand something of its scaling properties – for example, the extent to which scores may be summed to a single score (or if not, how it constituent items may be grouped), and the extent to which it could produce (or be transformed to produce) interval level data, suitable for mathematical manipulation.

The Medical Outcomes Trust has proposed a framework for evaluation of clinical measurement tools [10] with a defined set of review criteria. The key review questions most relevant to outcome measures for rehabilitation are listed in Table I. To ensure that a tool is widely taken up, however, there is a further important attribute – clinicians must *want* to use it. We have therefore added a new criterion, which we have called “Engagement”.

**Table I.** Key criteria for tools for inclusion within a national data set.

Criterion	Review question
Validity	Does the tool measure what in purports to measure? Face validity – in measuring the thing of interest Content validity – domains of interest are adequately represented Construct validity – relationships with other measures
Reproducibility	Do repeated applications of the measure produce the same results? Intra-rater reliability, intra-rater reliability (repeatability)
Scaling properties	Can individual item scores reasonably be added into a single total score? If not, how should they be grouped? To what extent does the scale provide interval level data?
Feasibility	Is the tool easy to apply in the clinical context?
Responsiveness	Does the tool detect changes when these occur? And does it remain stable when there is no change?
Interpretability	Do clinicians understand what the output from the tool means?
And …	
Engagement	Do clinicians actually *want* to use the tool?

Therefore, when choosing the measures that are to be incorporated into a national data set for rehabilitation, it is vital to ensure that the instruments are fit for purpose. They must not only have robust psychometric properties to support the generation of quantitative data, and be feasible to use in the clinical setting; they must also make a positive contribution by supporting clinical decision-making as an integral part of the rehabilitation process, so that they become a necessity for everyday clinical practice.

### Casemix and complexity

A universal challenge for clinicians is the ever-increasing pressure to manage more complex patients with less finance and resources. For services working within a limited budget, managing the complexity of the caseload becomes a key step in resource management. Across the world, governments are introducing standardised payment tariffs for health care with a view to driving up cost-efficiency. A variety of casemix systems have been developed to take account the differential costs of treating different patient groups.

Diagnosis is a poor indicator of cost in rehabilitation, where the major determinants of cost are staff time. Many of the international casemix models use global disability measures (such as the Functional Independence Measure (FIM [11] or the Barthel index [12]) not only as a measure of outcome but also as a proxy of needs for rehabilitation intervention. However, although these instruments correlate broadly with needs for care in hospital and community settings [13], they do not directly measure the requirement for nursing, therapy or medical intervention in terms of staff time. In addition, they focus primarily on physical care, and do not reflect the cognitive or psychosocial needs that are often most critical in patients with neurological illness or injury.

Casemix information is also required to interpret outcome data. Some patients can be predicted to do better than others, so clinical data sets must not only collect outcome measures but also the relevant information to identify key predictors of outcome. Age, severity of injury and co-morbidity are all well-recognised predictors, but we also know that higher intensity rehabilitation can improve outcome [14,15]. Many clinicians are well aware that they lack the resources to provide the level of input that is needed to optimise outcome, so it is also pertinent to record both the patient’s requirements for rehabilitation, and the extent to which these were met by the level of rehabilitation inputs provided. Purchasers of rehabilitation services may reasonably expect, however, that higher investment in intensive rehabilitation is justified by evidence for improved outcome. Thus, if we really want to understand what works best for which patients, clinical data sets must provide information at all three levels – needs, inputs and outcomes.

### The UK specialist rehabilitation outcomes collaborative (UKROC) database

The UKROC database has been set up to support the collection and collation of case-episode data for specialist inpatient rehabilitation services in the United Kingdom. The database has been established at Northwick Park Hospital through a National Institute for Health Research (NIHR) Programme Grant [16]. In the first 5 years, it will focus on neuro-rehabilitation and ultimately including data from all specialist neuro-rehabilitation services in England [8].

The UKROC data set development is undertaken in collaboration with the British Society of Rehabilitation Medicine (BSRM) and the Australasian Rehabilitation Outcome Centre (AROC), which is now 10 years into a similar programme [11]. Learning from their experience, as well as from other large international data collation programmes (such as the Uniform Data Systems [UDS] database in the United States), we have taken a novel approach to the type of data that are gathered. The AROC and UDS data sets rely principally on the FIM as both an outcome measure and a surrogate for case complexity. However, as noted above, the FIM has some limitations in both of these roles, so the UKROC team has developed a cluster of measures which are intended to provide more directly the information that service providers and planners require to develop effective and cost-efficient rehabilitation services, sensitive to the needs of their local population.

Systematically recorded for each admitted case episode, the UKROC data set will:

1. provide information on casemix and episode costs to inform the development of payment tariffs that are weighted for case complexity,
2. provide benchmarking information to support improvement of patient care,
3. serve to “open the black box of rehabilitation” by providing case-by-case information on rehabilitation requirements, inputs and outcomes (including cost-benefits) of rehabilitation for patients with different levels of need.

The tools within the data set have been established through an iterative process of development and evaluation over a period of 10–15 years.

The UKROC data set and represents the inpatient rehabilitation subset of the Long Term neurological Conditions data set^1^. The principal data items are summarised in Table II. In addition to demographic and process data, the 30-item data set also records:

**Table II.** Overview of UKROC data set.

Domain	Content
Demographics	Age, gender, ethnicity, funding authority etc.
	Diagnosis (ICD 10 code). Casemix category (HRG v 4 code)
Process	Response times – referral to admission
	Source of admission, interruption to treatment
	Length of stay
	Discharge destination
Needs (Complexity)	Rehabilitation Complexity Scale
Inputs	Northwick Park Dependency Scales: Nursing Dependency and Care Needs Assessment Therapy Dependency Assessment
Outcomes	Barthel Index
	FIM or UK FIM + FAM (+Neurological Impairment Set)
	Goal attainment scaling (GAS)

HRG, Healthcare Resource Group (The UK casemix classification is currently in version 4); ICD 10, International Classification of Disease version 10; FIM, Functional Independence Measure; UK FIM + FAM, UK Functional Assessment Measure.

1. The complexity of an individual’s rehabilitation *needs* – captured by the Rehabilitation Complexity Scale (RCS [17])
2. The rehabilitation *inputs* (nursing, therapy and medical) provided to meet those needs – captured through the Northwick Park nursing [18,19] and therapy Dependency Scales [20]
3. *Outcomes*, captured by a hierarchical set of outcome measures ranging from the Barthel Index [21] at the simplest level; through the FIM to the UK Functional Assessment Measure (UK FIM + FAM [22]) for the tertiary (Level 1 [23]) services, which provide rehabilitation for patients with complex rehabilitation needs, beyond the scope of their local specialist services. Offering a range of tools helps to minimise the burden of data collection for low cost/high volume local services (where the emphasis is mainly on achieving physical independence in basic self-care), while enabling the more specialist services to capture the subtler changes in cognitive and psychosocial function that are often their primary target for intervention. This particular set of standardised outcome measures was chosen because previous research has demonstrated that 95% of specialist rehabilitation units in the United Kingdom were already using one of other of them in their routine clinical practice [24]. Moreover, the relationship between them is known so that data from all three can be used to provide a common language data set at the level of the Barthel Index [25].

^1^The LTNC dataset may be downloaded from the NHS Information centre Website: http://www.ic.nhs.uk/services/datasets/document-downloads/long-term-neurological-conditions-ltnc-data-set.


Itemised scores are recorded at admission and discharge for all cases, and may also be recorded serially for highly complex patients to capture changing needs over time. Documenting the input actually provided to meet those needs supports the identification of unmet need, and also the reasons for variance. The chosen input measures provide a directly costable measure of rehabilitation intervention (based on calculation of staff time), as well as ongoing care, which allows the cost benefits of rehabilitation to be quantified in terms of long-term savings in ongoing care costs [26,27].

From the point of construction, the UKROC data set is designed to provide more directly than other data sets, the information that clinicians and service commissioners require. However, a further step is required – to make it accessible, so that clinicians not only *will* collect the data, but actually *want to*, for the added value that this provides to patient management at the “coal-face” of clinical care. Table III summaries the common barriers and some possible solutions to clinician engagement in outcome measurement that have been explored during development of UKROC. In the next section, we describe the practical steps that we have taken, within the design of the UKROC database and its implementation within our service, to apply those solutions and integrate data collection into our clinical decision-making process. We hope that this example will assist other services to find ways to implement the data set within their own context.

**Table III.** Common barriers and possible solutions to clinician engagement in outcome measurement.

Barrier	Problems	Possible solutions proposed by the UKROC programme
Time	Clinicians are increasingly hard pressed for time. Recording of outcome measures is typically deferred to the end of the day, relying on the clinicians to stay on after hours. Tools which require multidisciplinary scoring are particularly problematic because of the extra time needed to get clinicians together.	Tools must be as timely as possible to complete. If more complex tools are used, the additional benefits should justify the time invested to complete them. There should be dedicated time for completion. For measures that require multidisciplinary scoring, time should be allocated during routine multidisciplinary team meetings, to avoid the necessity of extra meetings.
Priority, leadership, and support	The priority for clinicians will always be clinical care. There is often a lack of leadership and drive to prioritise outcome measurement.	Strong leadership is required to ensure that measures are completed. This is a role for the consultant or other senior team member with string managerial influence. It should not be delegated to junior staff or external personal (such as research staff) – the drive must come from within the team itself.
Admin support and computerization	Administration support is often lacking so that computer entry is left to the clinical staff. Computer software is often unfriendly, leading to inaccurate data entry.	Administration support should be provided to enter the data. User-friendly data entry tools are required to ensure accurate completion, and staff who enter the data should have adequate training.
Relevance and usefulness	Outcome measures may seem to have little relevance to the client group or the objectives for rehabilitation. The tools are seen as just another chore, with no benefit to the team to reward their efforts in completion.	A range of outcome tools is required to ensure that the rehabilitation objectives for the group are reflected. Tools should offer added benefits that assist the clinician in their daily practice/clinical decision-making, so that they are perceived to be relevant.
User friendliness	Complicated, poorly presented tools can be off-putting. In particular, those with complicated scoring schemes and formulae are often poorly understood by clinicians.	Tools should be presented in a user-friendly manner – for example in verbal form that the user (e.g. clinicians, or patient for self-report tools) can relate to. The application of scores and formulae may then be applied when information is computerised.
Training and understanding	Clinicians are often required to complete outcome measures with little or no training. They often have little understanding of the use the data are put to, and are afraid that they will be misinterpreted.	Training in the use of outcome measures should be provided at a level appropriate to the individual’s role. Clinicians on the ground require training in the application of the tool. Senior staff should have a clear understanding of how to interpret the data that derives from the measure.

## Exemplar service setting

The Regional Rehabilitation Unit (RRU) at Northwick Park Hospital provides a tertiary (Level 1) post-acute inpatient specialist rehabilitation service for younger adults with severe complex neurological disabilities – including physical, cognitive, behavioural and/or communicative problems [28]. The service has a catchment of over 15 million population across the South-East portion of England, and takes a selected group of patients with highly complex rehabilitation needs. The 24-bed unit is one of a consortium of eight tertiary specialised neuro-rehabilitation services in London. It is situated within an acute district hospital, which supports the early admission of patients from neurosurgical and major trauma centres, at a stage when they still require high level specialist medical and nursing care (e.g. tracheostomy, ventilatory support). The guiding philosophy of the unit is to provide a coordinated interdisciplinary goal-orientated approach to rehabilitation. Goal setting involves patients and their families (to the extent of their ability) so that the rehabilitation programme is conducted in partnership between the patient and the treating team.

### Casemix management – the RCS

As noted above, managing the complexity of the caseload becomes a key step in resource management. The RCS is a simple 4-item scale (range 0–15) which records the level of care, nursing, therapy and medical needs of the patient. It is shown to be valid and simple to apply within the clinical setting, taking no more than 2–3 min to rate [29].

In the RRU, the RCS is recorded for all patients on the unit as part of the weekly ward multi-disciplinary round. The information is used in real time to identify the current casemix on the ward, and to plan admissions accordingly. The unit runs a waiting list of approximately 18–20 patients at any one time, and the majority of which have highly complex medical and nursing needs. The limiting factors for admissions are (1) bed capacity and (2) the requirements for nursing care. We have learned from experience that the nursing staff struggle when the number of patients with “heavy” nursing needs (RCS care and nursing scores of ≥5/6) exceeds 10 out of 24 beds. When the complexity profile of the ward is known, casemix can be adjusted by selecting admissions to balance the number of “heavy” patients with lighter cases, or by reducing the overall numbers to support a higher proportion of “heavy” patients, according to waiting-list pressures. Moreover, as the RCS can be collected prospectively, it is used to plan admissions appropriately 2–3 weeks in advance, and so to maintain a high level of occupancy by ensuring that incoming patients arrive as soon as the bed becomes vacant.

The serially recorded RCS is also used as the casemix tool to support reimbursement for the service according to a payment system that is weighted for complexity. The commissioning currency for the unit is a multi-level weighted bed-day tariff, which changes over time with the level of complexity [8]. So for example, a patient with very complex needs is reimbursed at the high rate during the first part of their admission. However, as they become more independent and their needs for care and nursing reduce, the tariff (which is adjusted according to the RCS score) falls. This reducing tariff provides an incentive to move patients on to other services (e.g. community rehabilitation) when they no longer require the highly specialised environment of the tertiary rehabilitation unit.

Because the RCS scores are used on a weekly basis to plan admissions, the staff find it clinically useful and are highly motivated to gather the information. Moreover, the explicit link between the RCS and reimbursement ensures that the hospital management provides the level of administrative support that is required to collect and collate the data.

### Rehabilitation inputs – the Northwick Park Dependency scales

The Northwick Park Dependency tools have been designed to provide a simple estimation of rehabilitation inputs in terms of staff time. Computer entry of the raw dependency scores into the customised UKROC software automatically generates the calculations of staff time together with a range of other clinically useful outputs that are described below.

- The nursing dependency scale (NPDS [18]) provides a simple assessment of care and nursing needs, which takes about 3–5 min to score by a nurse who knows the patient well. It translates by way of a computerised algorithm to the Northwick Park Care Needs Assessment [19], which provides an estimation of the total nursing and care staff hours.
- The therapy dependency tool (NPTDA [20]) is the therapy equivalent, which collates therapy inputs from the multidisciplinary team and also translates by a computerised algorithm into an estimation of therapy hours for each discipline (including medical staff). It takes 7–8 min to score.

Together these tools provide a practical means to record the relative proportion of staff time that is spent in managing patients with different levels of complexity (as rated by the RCS). In other words, they provide information that is similar to that provided by activity analysis, but in a much timelier manner. The calculations of staff time so derived have been used to calculate the differential costs within the weighted payment tariff [8]. Moreover, the tools may be applied both prospectively (to identify the needs for input) and retrospectively (to identify inputs actually provided) and to identify unmet need [20]. This, quantified over the entire caseload provides an estimate of the additional staff time (broken down by discipline) that would be needed to provide the clinically appropriate level of intervention for a given casemix, and has been used in our service to make the case for additional resources to meet the demands of an increasingly complex caseload.

However, in addition to providing information about the overall time spent with a given patients, the tools also provide useful information about the *type of interventions*, which the clinical team uses for a variety of purposes in the delivery of the rehabilitation programme.

The NPDS records 16 items of basic care needs and a further seven describing special nursing needs that require input from a qualified nurse. Importantly, it includes time spent in communication, behavioural management, and maintaining safety, which can take up a considerable proportion of staff time, but are not identified by the FIM or Barthel Index. Entering NPDS data into the UKROC software automatically generates the NPCNA which provides practical added value to the NPDS.

- It produces a printable timetable of care needs in the community, which is routinely used in discharge planning meetings on the RRU to inform the provision of a suitable ongoing care package [19].
- It generates a summary of the serial scores for the programme, which are included in the patient’s discharge summary.
- It also calculates an estimated cost of community care, and this information has been used to demonstrate the cost-efficiency of rehabilitation in reducing the long-term costs of care [26].

The fact that this practical information can only be obtained by entering the NPDS data ensures that the NPDS scores (which are rated fortnightly for all patients) really do get into the database, where they are collated for future analysis.

The NPTDA is similarly rated by the therapy team at fortnightly intervals for all patients, and computerised data entry is required to get the maximum benefit from the tool. Like the NPDS, the NPTDA provides more detailed information about the type of interventions provided, and also supports the identification of very high intensity inputs on the occasions when these have been required [20].

- The tool records both “direct” interventions in contact with the patient and “indirect” patient-related interventions away from the patient (e.g. report-writing, team meetings, or chasing-up equipment). Patients and their families are sometimes unaware of the amount of time that may be required for indirect activities, especially around the time of discharge, so the collation of this information can be helpful for explaining to them why the amount of direct contact time is often reduced in the run-up for discharge.
- The NPTDA also provides information that is useful for the therapy managers to ensure equitable input for patients in accordance with need. Item 17 on the NPTDA records “Emotional Load on Staff”. This item is included in the recognition that, for a variety of different reasons, working with some patients can be extremely draining for members of the team. The formal identification of emotional load acts an early warning system and allows therapy team leaders to ensure that high-scoring patients are shared out by the team, to avoid excessive burden for individual members of the team and particularly to support the more junior members of staff.

## Outcomes evaluation

The RRU team holds a monthly discharge review meeting for reflecting briefly on each patient discharged from the unit in the previous month. As part of discharge planning, the patient and their family are asked to provide feedback about their experience on the unit under three main headings:

- What went well during the programme?
- What went not so well? and
- What lessons could be learned for the future?

The treating team also provides reflection under the same headings, and the information is combined together with a review of the outcome measures [30]. This feedback loop helps to sustain clinicians’ behaviour, as they can actually see the changes recorded through measurement.

### Functional assessment measure (UK FIM + FAM)

The primary standardised outcome measure of the unit is the UK FIM + FAM [22]. This is a 30-item scale comprising the 18-item FIM, together with 12 additional items to extend the evaluation of psychosocial function. The RRU is the UK national centre for training and operationalisation of the UK FIM + FAM, so all staff are routinely trained in its use.

**Figure 1.**
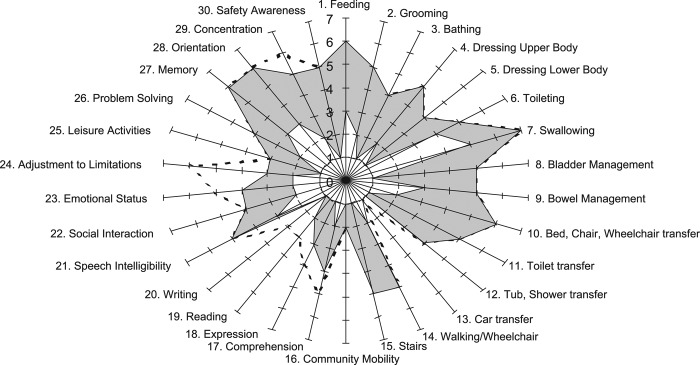
Example of a FAM-Splat for a patient with traumatic brain injury. The FAM-Splat provides graphic presentation of the disability profile in a radar chart. The 30 items are arranged as spokes of the wheel and the levels from 1 (total dependence) to 7 (total independence) run from the centre outwards. Thus a perfect score would be demonstrated as a large circle. The shaded area shows the change between admission and discharge for each item. The dotted line indicates where a goal score set at admission was not achieved.

In this unit, each patient has an “admission FIM+FAM score” recorded within the first 10 working days from admission, together with a “goal” scores – that is the score level for each item that the team expects the patient to have at discharge. A discharge FIM + FAM rating is then recorded within the last 7 days before discharge, without reference to the admission and goal scores. All three ratings are entered into the UKROC software, which generates a graphic presentation in the form of a “FAM-splat” or radar chart, illustrated in Figure 1 [31]. The FAM-splat shows at a glance the areas in which the patient has and has not made functional gains. It also shows the areas in which they fell short of, or exceeded, the team’s expectations. This provides an opportunity for the team to discuss the reasons for variance from expected levels, and serves to enhance team learning about what can and cannot be achieved. Once again the requirement for computerisation to generate the FAM-splat in time for the meeting ensures that the ratings are entered into the database before each patient is discharged from the unit, and so supports the completeness of data collection.

## Goal attainment scaling

While standardised measures, such as the FIM and/or FAM, provide general information regarding functional gains that can be used to compare outcomes across different services and populations, they have recognised floor and ceiling effects, and do not necessarily capture the intended aims of the programme or the gains that that are most important to the patient. As the rehabilitation programme is centred on goals that are set with the involvement of patients and their families, goal attainment scaling (GAS) provides an opportunity to record the extent to which those personal goals were achieved. GAS is not in itself a measure of outcome, but a measure of the achievement of expectation. It does not replace the need for standardised outcome measures but, recorded alongside them, it provides an important person-centred aspect to the evaluation of outcome, as well as an opportunity to capture the more diverse benefits of rehabilitation [32].

GAS was first introduced in the 1960s by Kiresuk and Sherman [33] originally in the context of education for people with learning disabilities. There is now a large literature on the use of GAS in rehabilitation, and the approach has strong protagonists and equally strong antagonists [34]. In clinical practice, although many clinicians have welcomed an approach which reflects the achievement of individual patient goals, the uptake of GAS in routine clinical practice has been limited by the rather cumbersome process for implementation as recommended by the originators.

- Within GAS, the level of goal attainment is rated on a 5-point scale ranging from “–2” (much less than expected) to “+2” (much more than expected). Achievement of the expected level scores “0”. Many clinicians are put off by the zero and minus ratings which they believe to be de-motivating for patients.
- To improve the rigor of assessment, Kiresuk and Sherman recommend the use of a “follow-up guide” with pre-defined descriptors set for each of the five levels for every goal [35]. This is very time-consuming and is essentially not feasible in the context of a busy ward or clinic.
- If the baseline score is set at “–1” to allow for the possibility of deterioration as well as improvement, there is no rating to reflect partial achievement of a goal [36].
- Clinicians are also sometimes daunted by the complicated looking formula which is used to calculate a composite GAS T-score.

Within UKROC, we have developed a simpler, more user-friendly approach to GAS, which we have called the “GAS-light” model, or “GAS without tears” [37] for use within the clinical setting. In this model, we have sought to “de-myth” GAS. It is simply applied as an integral part of the normal clinical process for goal-setting and review, without the need for additional steps, other than simply entering the information into the UKROC software.

- Instead of pre-defining all five levels, the team draws up a SMART definition just for the expected level of achievement (0 score). The level of achievement is then rated by the patient and team at the end of the programme, using the expected level definition at the reference point.
- Instead of using the −2 to +2 numeric scale, clinicians are presented with a 6-point verbal rating scale, which includes the option to record partial achievement.
- This is then converted back to the 5-point numeric scale by the computer software, which also automatically calculates the GAS T-Score.

Some authors have advocated the need for an independent assessor to evaluate goal achievement [38]. However, we believe that this is not only unnecessary, but actually undermines some of the benefits of GAS. Negotiating and agreeing realistic (but suitable challenging) goals forms part of the important educational process, which will develop the patient’s skills in setting and monitoring their own goals autonomously after they leave the programme. Collaborative goal-setting and review, with involvement of the patient and/or family, is a key component of the patient-team partnership, and a critical part of this goal management training [39], especially for patients who have problems with executive function. In our unit, we have developed an aphasia-friendly picture-based form of GAS to assist the involvement patients with cognitive and/or communication deficits in their own goal setting and review.

### Leadership, generalisability, and future direction

The UKROC database is now in its third year of data collection. Currently, 44 out of an estimated total of 60 specialist neuro-rehabilitation units are submitting at least the minimum data set for all clinical episodes on a regular basis. The number of services is growing, year on year, as is the quality and completeness of the data. Clearly, this is an early stage in the developmental process. We anticipate that, as data collection becomes embedded in clinical practice, the data set will expand over time to encompass other tools that will assist rehabilitation clinicians to improve the quality of patient care, including an expanded range of self-report measures to evaluate the longer impact of rehabilitation on their daily lives. However, the achievement of a consistent core set of clinical data is an essential first step, and even that presents a considerable challenge for many services at the current time.

Key steps to the successful integration of data collection within our exemplar service have been the development of user-friendly software (which is an ongoing process) and embedding the use of the tools into the everyday clinical decision making. However, the other principal requirements for successful integration of data gathering are staff training and ongoing commitment from the senior staff and managers. This is critical to the maintenance of effort required to provide assurance of data quality in the longer term.

While the tools may be collected on the ground by all members of the interdisciplinary team, strong leadership is required to ensure that the instruments are completed at the right time and entered into the database. We consider this to be a role for the consultant. On our unit, the weekly multidisciplinary team meeting is used as the focus point to gather together all the relevant RCS, NPDS and NPTDA scores for that week, and these are reviewed for completeness by the unit’s consultant, before passing them back for data entry, which ensures that missing data are kept to an absolute minimum. The UKROC data monitoring team reports that other services with a good record for complete data submission, also tend to be those with strong consultant leadership.

The commitment of services commissioners is also helpful. In a separate article in this issue, we demonstrate how the UKROC data set has been used to engage service managers and commissioners to make the case for resources to improve patient care [40]. In London, the Specialised Commissioning Consortium now requires the submission of the complete data set for each patient as a condition for payment. All of these serve to ensure that the data are collected and verified in real-time, and not just as a retrospective exercise. From 2011/12, the UKROC database will be commissioned under a service level agreement to provide the commissioning data set for all eight Level 1 services in London, and in future will become the national vehicle for collation of activity data to apply the multi-level payment model and calculate re-imbursement. This will help to ensure that the database is sustained after the end of the initial research-based funding, in a manner analogous the Australian system [11].

## In summary

By integrating the use of the data set measures in everyday clinical practice and making the data “live” for clinicians on the ground we have succeeded in engaging their hearts and minds in the task of data collection, and thereby have achieved a very high rate level of consistency in data recording. However, staff training and ongoing commitment from senior staff, managers, and commissioners are critical to the maintenance of effort required to provide assurance of data quality in the longer term.

## Copies of instruments

Enquiries about any of the tools mentioned in this article should be directed to the corresponding author. Regular training workshops for the FIM+FAM and GAS are run by the UKROC team at Northwick Park Hospital. The RCS, the NPDS and NPTDA and other useful resources are available free of charge (Please see website: http://www.ukroc.org).

## Ethics approval

The Regional Rehabilitation Unit gathers these outcome data routinely in the course of clinical practice. Research Ethics Committee permission has been obtained to report the data retrospectively for research and audit purposes.
